# Comparative transcriptome analysis provides insights into grain filling commonalities and differences between foxtail millet [*Setaria italica* (L.) P. Beauv.] varieties with different panicle types

**DOI:** 10.7717/peerj.12968

**Published:** 2022-02-18

**Authors:** Hui Song, Tao Wang, Long Li, Lu Xing, Hui fang Xie, Bai li Feng, Jin rong Liu

**Affiliations:** 1 Anyang Academy of Agriculture Sciences, Anyang, Henan, China; 2 College of Biology and Food Engineering, Anyang Institute of Technology, Anyang, Henan, China; 3 Innovation and Practice Base for Postdoctors, Anyang Institute of Technology, Anyang, Henan, China; 4 College of Agronomy, Northwest A&F University/State Key Laboratory of Crop Stress Biology for Arid Areas, Yangling, Shaanxi, China

**Keywords:** Foxtail millet, Grain filling, Transcriptome, Panicle type

## Abstract

Grain filling affects grain weight and quality and is among the most critical factors in determining the yield and quality of cereal crops. Though hybrids have larger panicles and numerous spikelets with a larger sink capacity than conventional varieties, data on the grain filling commonalities and differences between foxtail millet varieties with different panicle types remain sparse. In this study, we found that “Zhang Gu 13” (ZG, large panicle) exhibits a significantly higher panicle weight than “Yu Gu 18” (YG, conventional panicle) at the early stage of grain filling, but the weight of YG increased rapidly and gradually overtook ZG during the middle stages. A temporal expression pattern analysis demonstrated that the genes involved in photosynthesis, metabolic pathways, and phenylpropanoid biosynthesis were downregulated, while those related to peroxisome function, purine metabolism, and zeatin biosynthesis were upregulated during grain filling in both varieties. A total of 6,832 differentially expressed genes (DEGs) were identified in both varieties, with the majority identified at the early and late stages. A Kyoto Encyclopedia of Genes and Genomes (KEGG) pathway analysis further revealed that the upregulated DEGs in YG were associated with gibberellin (GA) biosynthesis, ATP-binding cassette (ABC) transporters, and plant hormone signal transduction. Photosynthesis-related DEGs, such as photosystem and antenna proteins, were significantly upregulated in ZG. This study provides preliminary insights into the differences in gene expression and molecular mechanisms of grain filling between ZG and YG in the North China summer-sowing region.

## Introduction

Cereal crops are invaluable sources of food, animal feed, and fuel globally. Hence, breeding for high-yielding varieties has always been a major goal in crop improvement programs. Grain development strongly affects cereal crop yield. The primary stage of grain development, the grain filling process, is characterized by a rapid increase in grain weight due to starch accumulation. As it determines the grain size, yield, and quality of cereals (Saini & Westgate, 1999; Zhou, Yin & Xue, 2013), improving grain filling has become essential in breeding for good quality and high yielding varieties (Jin et al., 2013).

The change in plant hormone levels strongly influences grain filling. For instance, increasing the cytokinin (CK) content in caryopsis could improve the grain filling of rice varieties with large panicles, thereby increasing their yield (Panda et al., 2018). Grain filling of rice inferior spikelets (later-flowering spikelets which are located on proximal secondary branches) is regulated by abscisic acid (ABA) through proteins and phosphoproteins participating in carbon, nitrogen, and energy metabolism (Zhang et al., 2012a). In wheat, *TaGW2-6A* regulates gibberellin (GA) synthesis *via* GA 3-oxidases (GA3OX), leading to higher expression of *GASA4*, which controls cell elongation and division of the endosperm during grain filling (Li et al., 2017). Moreover, applying exogenous auxin (IAA) in caryopses located on proximal secondary branches before fertilization promotes gene expression, caryopsis development, and grain weight (Jia et al., 2021). Finally, salicylic acid (SA) mediates grain filling by upregulating the abundance of proteins involved in glycolysis, the tricarboxylic acid (TCA) circle, starch and sucrose metabolism, and oxidative phosphorylation under dry soil conditions (Kimbembe et al., 2020).

The activities of enzymes involved in sucrose-starch conversion also affects the rate of grain filling. Sucrose synthase activity is vital in regulating biosynthesis and in the accumulation of starch in rice grains. It can thus be used as an index for high-yielding rice (Counce & Gravois, 2013). Similarly, decreased fructose-6-phosphate 1-phosphotransferase (PFPase) activity results in compromised carbon metabolism, increased soluble sugar content, and abnormal starch biosynthesis (Duan et al., 2016). Low ADP-glucose pyrophosphorylase (AGPase) activity in *gif2* rice mutants causes slower grain filling, resulting in low grain weight and yield compared to the wild type (Wei et al., 2017). Moreover, cell wall invertases are responsible for the sucrose unloading process, which regulates the final grain weight and starch content in rice (Wang et al., 2008).

Numerous pivotal genes involved in grain filling in major crops have been identified. For instance, *OsNAC127* and *OsNAC129* affect grain development by regulating sugar transportation in rice (Ren et al., 2021). In addition, the rice G protein γ subunit, DEP1/qPE9–1, positively regulates grain filling by increasing the auxin and CK content of grain (Zhang et al., 2019). Similarly, ZmBES1/BZR1-5 could improve the grain size and weight in maize through inhibiting the transcription of AP2/EREBP genes by binding to E-box and BRRE elements (Sun et al., 2021). In wheat, the endosperm-specific transcription factor, *TaNAC019*, regulates glutenin and starch accumulation, consequently improving grain quality (Gao et al., 2021).

Foxtail millet [*Setaria italica* (L.) P. Beauv.], which originated in China, has become an important food and feed crop in arid and semi-arid regions in both Asia and Africa (Lata, Gupta & Prasad, 2013). It is emerging as a promising model for functional genomics amongst the Panicoideae, especially for C4 photosynthesis studies (Doust et al., 2009). The sequencing and publication of its genome have promoted molecular and functional genomics studies of this crop (Zhang et al., 2012b; Bennetzen et al., 2012; Jia et al., 2013; Zhao et al., 2020). Nonetheless, the molecular mechanisms of grain development and filling in foxtail millet remain unclear (Xiang et al., 2017; Wang et al., 2020).

In this study, we used RNA-sequencing to probe the gene expression changes associated with grain filling between the foxtail millet varieties Yu Gu 18 (YG) and Zhang Gu 13 (ZG) at six distinct grain development stages. YG has the advantages of high yield and broad adaptability and is one of the key varieties promoted for cultivation in China. In the summer sowing season in the Northern China, the seed setting rate (percentage of grain weight per panicle) of this accession is 81.68%, and the average yield is 5,700 kg/hm^2^. ZG is a popular hybrid with a stable yield and the main accession cultivated used in the Northwest spring-sowing region, with a seed setting rate of 75.6% and an average yield of 5,250 kg/hm^2^ (Fan et al., 2019; Song et al., 2020). Here, we provide new insights regarding differences in the molecular mechanisms underlying grain filling in these two foxtail millet accessions with different panicle types. Ultimately, our study provides an important molecular resource for foxtail millet yield and quality improvement.

## Materials and Methods

### Plant materials and sample collection

The varieties used in our study were “Yu Gu 18” and “Zhang Gu 13”, grown in the experimental station of Anyang Academy of Agriculture Sciences (North China summer-sowing region, N 36°18′, E 114°37′, altitude 75 m) from May to October 2020. A randomized block design was used to perform the field experiments, with 20 m^2^ for each plot and each plot receiving an appropriate supply of fertilizer and water during the growth period. Fertilizer was applied in the following amounts: N: 544 kg/hm^2^, P_2_O_5_: 489 kg/hm^2^, K_2_O_5_: 77 kg/hm^2^. Starting 7 days after anthesis, same-aged panicles of similar sizes were harvested every 7 days, up to day 42 (T1–T6). Five panicles were dried at 105 °C for 0.5 h and 70 °C until their weight was constant, respectively. Panicles were weighed at the T1, T2, T3, T4, T5, and T6 stages of development. The significance among the means of panicle weight was verified using ANOVA, and mean differences were compared by Fischer’s least significant difference (LSD) test (*p* < 0.05).

### RNA extraction, library construction, and sequencing

The total RNA of the kernels of each panicle was extracted using TRIzol Reagent (Ambion, Inc., Austin, TX, USA) according to the manufacturer’s protocol. RNA purity and yield were evaluated using the NanoDrop 2000 spectrophotometer (Thermo Scientific, Waltham, MA, USA). RNA integrity was assessed using the Agilent 2100 Bioanalyzer (Agilent Technologies, Santa Clara, CA, USA). The libraries were then constructed using the TruSeq Stranded mRNA LT Sample Prep Kit (Illumina, San Diego, CA, USA) according to the manufacturer’s instructions. The RNA was sent to Shanghai OE Biotech for library construction and 125 paired-end sequencing using an Illumina HiSeq 2500 (Illumina, San Diego, CA, USA). All sequence data were deposited into the NCBI database (Accession number: PRJNA721299, SAMN18713804 (YG), SAMN18713832 (ZG)).

### Data quality control and read mapping

Deep sequencing data were processed using Trimmomatic version 0.36 (Bolger, Lohse & Usadel, 2014). Clean reads were obtained by removing the low-quality sequences. Hisat2 version 2.2.1.0 (Kim, Langmead & Salzberg, 2015) was subsequently used to map the clean reads to the foxtail millet reference genome version 2.2 (*Setaria italica* v2.2: https://phytozome.jgi.doe.gov/pz/portal.html#!info?alias=Org_Sitalica).

### Temporal expression patterns of genes

We employed the Short Time-series Expression Miner (STEM) software (Carnegie Mellon University, Pittsburgh, PA, USA) to analyze the temporal expression patterns of genes during grain filling. The gene expression data of each sample was imported (three replications), and the default settings of the STEM program were used. A hyper geometric distribution test was also carried out to identify the significantly enriched profiles (*p* value < 0.05) (Ernst & Bar-Joseph, 2006).

### Differential expression analysis of genes

Principal component analysis (PCA) was performed to assess variability between the two assessed accessions using the PRCOMP command with default setting in the R-software package. The gene expression values were calculated as fragments per kilobase per million reads (FPKM), using Cufflinks version 2.2.1 (Trapnell et al., 2010). Differentially expressed genes (DEGs) were identified using the DESeq version 1.18.0 software with the R package functions ‘estimateSizeFactors’ and ‘nbinomTest.’ A gene was deemed differentially expressed with the absolute value of fold change > 2 and a *p*-value < 0.05 (Anders & Huber, 2012). The Venn and UpSet diagrams were drawn using TBtools with default settings (Chen et al., 2020).

### Functional annotation of genes

All DEGs were subjected to a Gene Ontology (GO, http://www.geneontology.org/) (Kanehisa et al., 2008) and functional enrichment analysis to assign their putative function. In addition, we performed a Kyoto Encyclopedia of Genes and Genomes (KEGG) analysis (http://www.genome.jp/kegg/pathway.html) (Ashburner et al., 2000) to map DEGs to the specific pathways that each gene is specifically involved in using the Blast_v2.2.26 software.

### Validation of RNA sequencing data

The expression profiles of 13 randomly selected genes were confirmed through qRT-PCR (Table S1). The RNA of the kernels was extracted using TRIzol reagent according to the manufacturer’s protocol (Tiangen, Beijing, China). The Primer-BLAST online NCBI tool was used to design primers specific to each DEG. The cDNAs were synthesized using a FastKing RT kit (Tiangen, Beijing, China) and qRT-PCR was performed following the protocol of the SYBR Green PCR Master Mix Reagent (SuperReal PreMix Plus, Tiangen, China) on an ABI 7500 fast Real-Time PCR System (Applied Biosystems, Waltham, MA, USA). Seita.7G294000 (*Actin 7*) was used as the internal reference (Qin et al., 2020; Yu et al., 2020) and the 2^−∆∆Ct^ method was used to calculate the relative expression level of each assessed gene (Livak & Schmittgen, 2001).

## Results

### Panicle weight variations during grain filling

**Figure 1:**
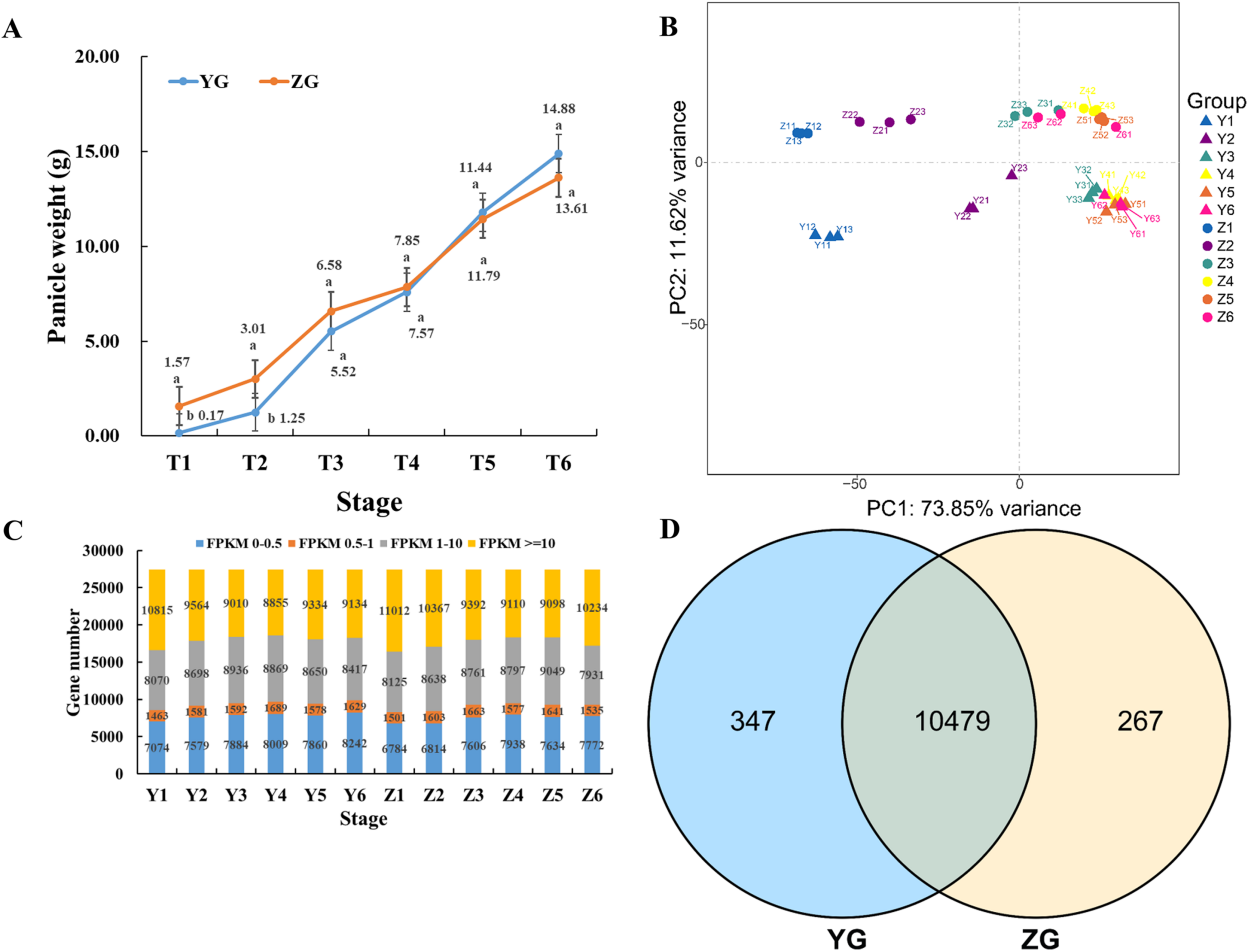
Overview of the panicle weight and transcriptomes of the two assessed accessions at different stages during grain filling. (A) Line charts showing changes in panicle weight. Error bars represent the standard error of the mean (±) (*n* = 3, n represents the biological replicates). Different letters indicate a significant difference between the two accessions at the 0.05 level. (B) Principal Component Analysis of the genes identified from the 36 samples analyzed by RNA-seq. Y11–Y13 represents the three biological replicates of Y1, and Z11–Z13 represents the three biological replicates of Z1. (C) Distribution of genes with different expression levels during each developmental stage in the two assessed accessions. (D) Venn diagram of the expressed genes *via* comparison of the two assessed accessions.

Panicles were harvested at six different time points (T1–T6) to compare the changes in panicle weight between the two assessed foxtail millet accessions. Compared to the YG accession, the panicle weight of ZG plants was significantly higher during the early stages (T1 to T3) of reproductive development (Fig. 1A). However, the panicle weight of YG increased rapidly and exceeded that of ZG during the late stages (T4 to T6) of panicle development (Fig. 1A).

### Transcriptome analysis of YG and ZG

Thirty-six libraries were constructed from the six assessed developmental stages for RNA-seq to obtain the gene expression dynamics of the two assessed foxtail millet accessions during grain filling. A total of 904 and 1,026 million raw reads were obtained for YG and ZG, respectively (Table S2). Subsequent removal of the adaptor sequences and low-quality sequences yielded 887 and 1,005 million clean reads with a Q30 base percentage higher than 94.89% and 94.38% for YG and ZG, respectively. Read mapping revealed 89.94% of YG reads mapped to the genome, with a unique mapping rate of 83.86%. Similarly, the mapping and unique mapping rates of ZG reached 91.81% and 88.08%, respectively.

The relative global relationships of biological replicates and clustering patterns of the samples were assessed through principal component analysis (PCA). Three samples of each time point clustered together (Fig. 1B). YG samples were separated from ZG samples, independent of the different developmental stages, suggesting that the genetic backgrounds are indeed different.

Normalized read counts for each gene was calculated and divided into four groups based on their expression level (Fig. 1C). Genes identified in all stages of the same accession were combined, and a Venn diagram was used to reveal the genes that were commonly expressed between the YG and ZG (Fig. 1D). Notably, there were 10,479 commonly expressed genes between the YG and ZG during the six assessed time points as part of the grain filling process. Moreover, there were 347 genes that were only expressed in YG, and 267 expressed genes that were only identified in ZG.

### Gene expression analysis of YG and ZG

**Figure 2:**
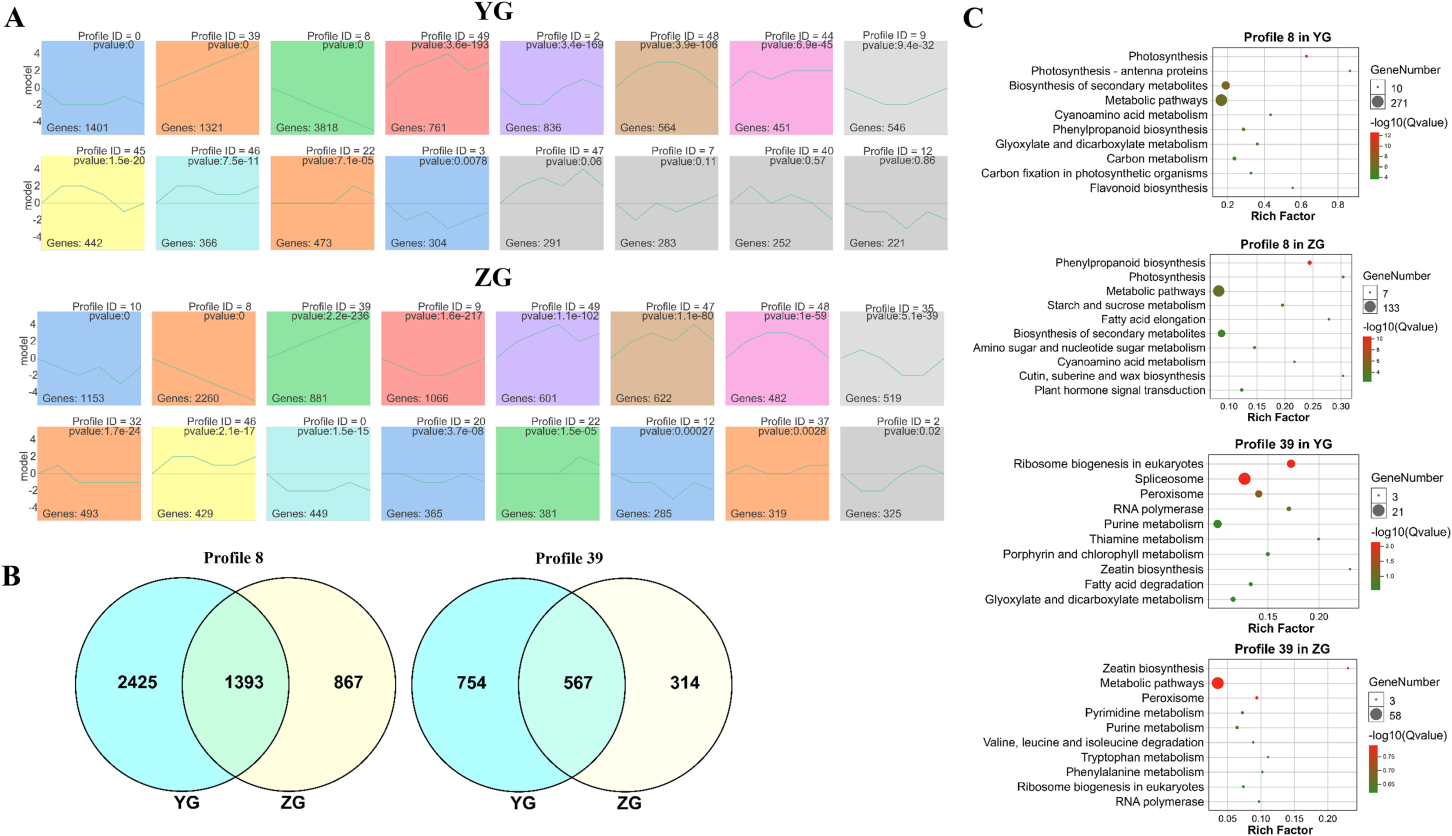
Expression trend and functional analysis of expressed genes during grain filling in the two assessed accessions. (A) Different expression patterns of expressed genes in the two assessed accessions. The top number represents the *p*-value. The bottom number represents the total number of genes in each category. The line represents the gene expression trends of each profile. (B) Unique and common genes between the two assessed accessions of profiles 8 and 39. (C) The top 10 KEGG pathways of genes in profiles 8 and 39.

The STEM (Short Time-series Expression Miner) software was used to cluster the genes which were expressed across the grain filling process in the two assessed accessions to determine their temporal expression patterns. Twelve and fifteen significantly enriched profiles (*p*-value < 0.05) were identified in YG and ZG from fifty profiles, respectively. Genes with similar expression trends were grouped into the same profile. Two particular model profiles (profile 8, downregulated profile; profile 39, upregulated profile) were identified (Fig. 2A). Venn diagrams revealed that both profiles had numerous uniquely expressed genes (Fig. 2B), suggesting that each accession utilises a unique regulation network during the grain filling progress. A KEGG enrichment analysis of profile 8 and profile 39 genes in both accessions revealed that genes involved in photosynthesis were downregulated in YG across the grain filling process, while those involved in ribosome biogenesis and the spliceosome were upregulated. Figure 2C shows the top 10 enriched pathways. In ZG, genes involved in starch and sucrose metabolism, phenylpropanoid biosynthesis and photosynthesis were downregulated, while those involved in peroxisome function and zeatin biosynthesis were upregulated across the grain filling process.

**Figure 3:**
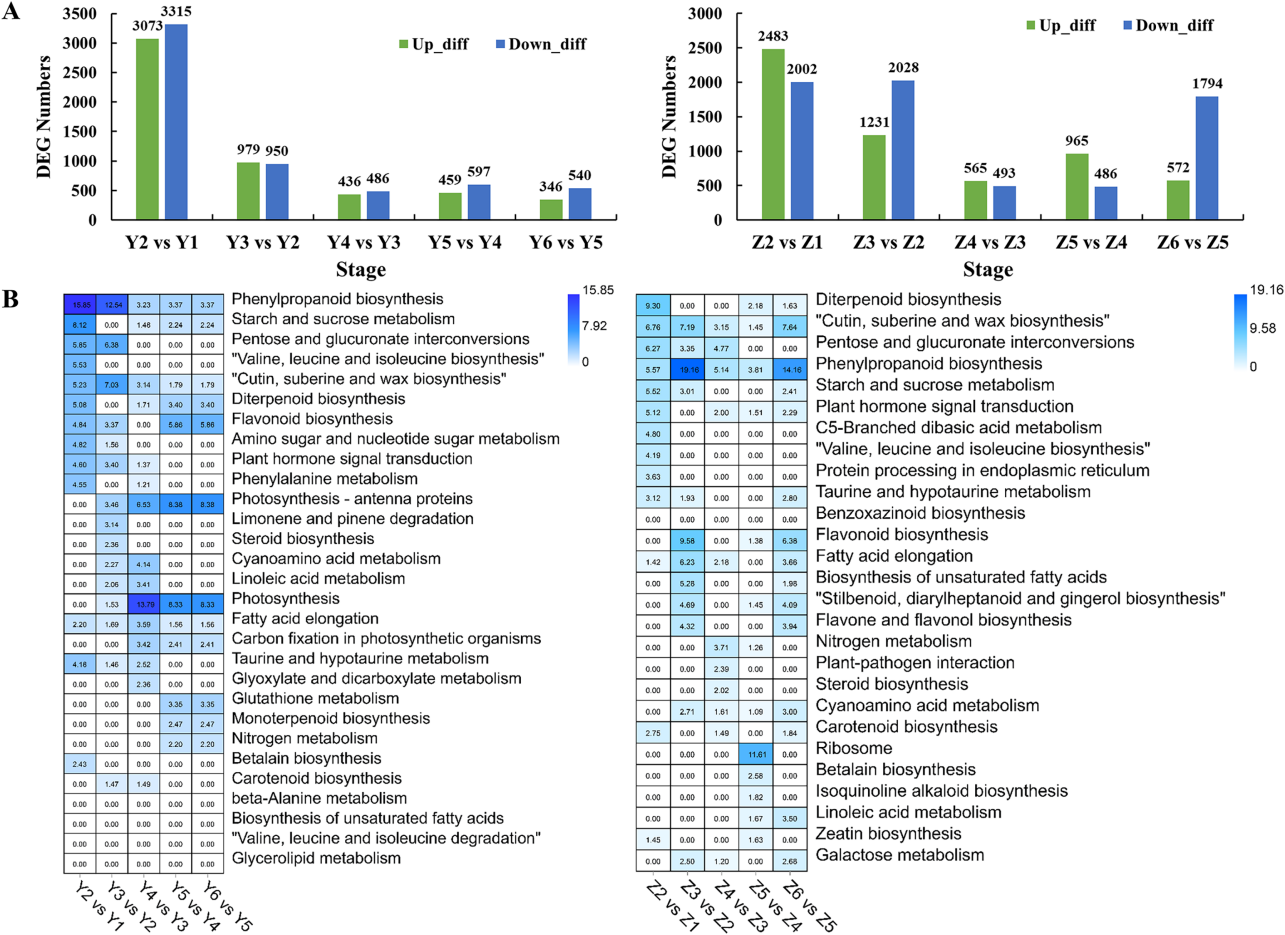
Numbers and functional analysis of DEGs between two adjacent stages in the two assessed accessions. (A) The number of upregulated and down-regulated genes between the two adjacent stages in the two assessed accessions. (B) KEGG pathway analysis of DEGs between the two adjacent stages in the two varieties.

Pairwise comparisons were performed to investigate the transcriptome dynamics during grain filling in YG. We identified 6,388, 1,929, 922, 1,056 and 886 DEGs in different YG comparison sets (Fig. 3A). A Gene Ontology (GO) enrichment analysis of the DEGs was performed to analyze the DEGs based on their biological process (BP), cellular component (CC), and molecular function (MF). Figure S1 shows the top 10 BP, CC, and MF GO enrichment terms of each comparison. Figure 3B shows the significantly enriched pathways among the different comparison sets. Similarly, there were 4,485, 3,259, 1,058, 1,451 and 2,366 DEGs identified in different comparison sets of ZG (Fig. 3A). Figure S2 shows the top 10 BP, CC, and MF GO enrichment terms of the DEGs during grain filling in ZG. Figure 3B shows the significantly enriched pathways among different ZG comparison sets.

### Identification of DEGs between YG and ZG

**Figure 4:**
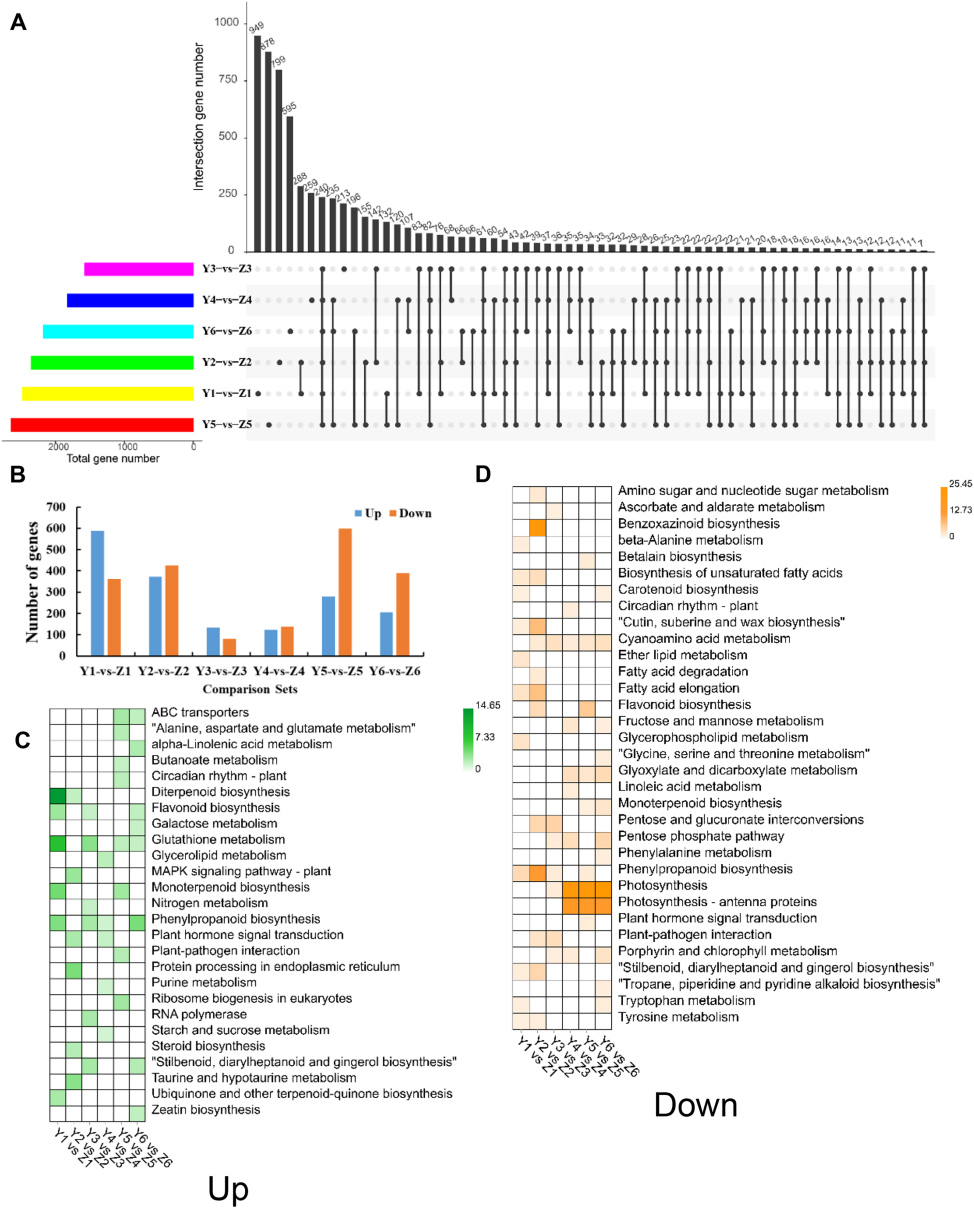
Overview of DEGs between the two assessed accessions at each stage. (A) UpSetR plot showing the overlapping of the specific DEGs between the two assessed accessions at each stage. (B) The numbers of upregulated and down-regulated genes between the two assessed accessions at each stage. (C) KEGG pathway analysis of upregulated DEGs at each stage in YG compared with ZG. (D) KEGG pathway analysis of down-regulated DEGs at each stage in YG compared with ZG.

We identified 6,832 unique DEGs between YG and ZG (Data Set S1) to help decipher the difference between the two assessed accessions during the grain filling process. The unique and common DEGs between YG and ZG were subsequently identified using an UpSetR plot. T1 had the highest number of DEGs, with 587 upregulated and 362 downregulated genes in YG compared with ZG. T3 had the lowest number of DEGs, with only 133 upregulated and 80 downregulated genes in YG compared with ZG (Figs. 4A and 4B). The upregulated DEGs were annotated to 50 GO terms: with 22, 15 and 13 of these falling into the BP, CC and MF subcategories, respectively. Cellular process, single-organism process, and metabolic process were the top three classes in the BP category. Cell, cell part, and organelle were the top three classes in the CC category. Binding, catalytic activity, and nucleic acid binding transcription factor activity were the top three classes in the MF category. Similarly, the downregulated DEGs were annotated to 55 GO terms: with 24, 18 and 13 of these falling into the BP, CC and MF subcategories, respectively (Fig. S3). Cellular process, metabolic process, and single-organism process were the top three classes in the BP category. Cell, cell part, and organelle were the top three classes in the CC category. Binding, catalytic activity, and transporter activity were the top three classes in the MF category. Figures 4C and 4D show the significantly enriched pathways of the DEGs in YG and ZG at each stage of the grain filling process.

### DEGs related to ATP-binding cassette (ABC) transporters

**Figure 5:**
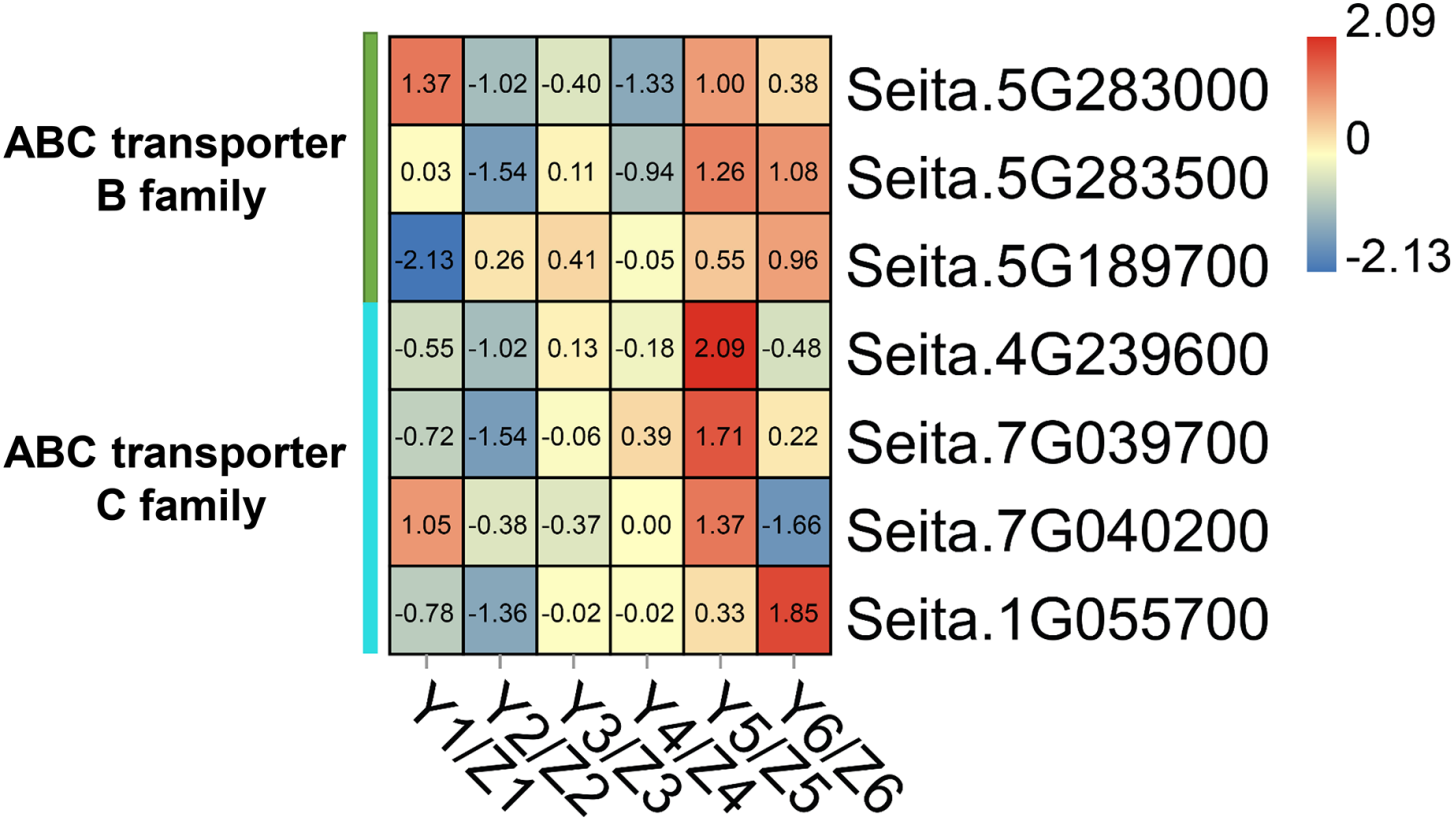
Heatmap analysis of genes associated with ABC transporters during grain filling in both assessed accessions. The value of Log2 (FPKM in YG/FPKM in ZG) is represented using color depth; red and blue represent the upregulated and down-regulated YG genes compared to those in ZG, respectively.

The ‘ABC transporter’ pathway was significantly enriched in the upregulated DEGs in the YG accession at late stage of grain filling (T5 and T6). Seven DEGs were related to ABC transporters which in the YG accession had a significantly higher expression than they did in the ZG accession (Fig. 5). In YG, Seita.5G283000 (*ABCB1-like*) and Seita.7G040200 (*ABCC1-like*) showed a higher expression at the T1 and T5 stages of grain development; Seita.5G283500 (*ABCB1-like*) was significantly upregulated at the T5 and T6 stages of grain development; Seita.4G239600 (*ABCC8-like*) and Seita.7G039700 (*ABCC1-like*) had higher expression levels at the T5 stage of development; and Seita.5G189700 (*ABCB1-like*) and Seita.1G055700 (*ABCB1-like*) showed their highest degree of expression at the T6 stage of development.

### DEGs related to the plant hormone signal transduction pathway

**Figure 6:**
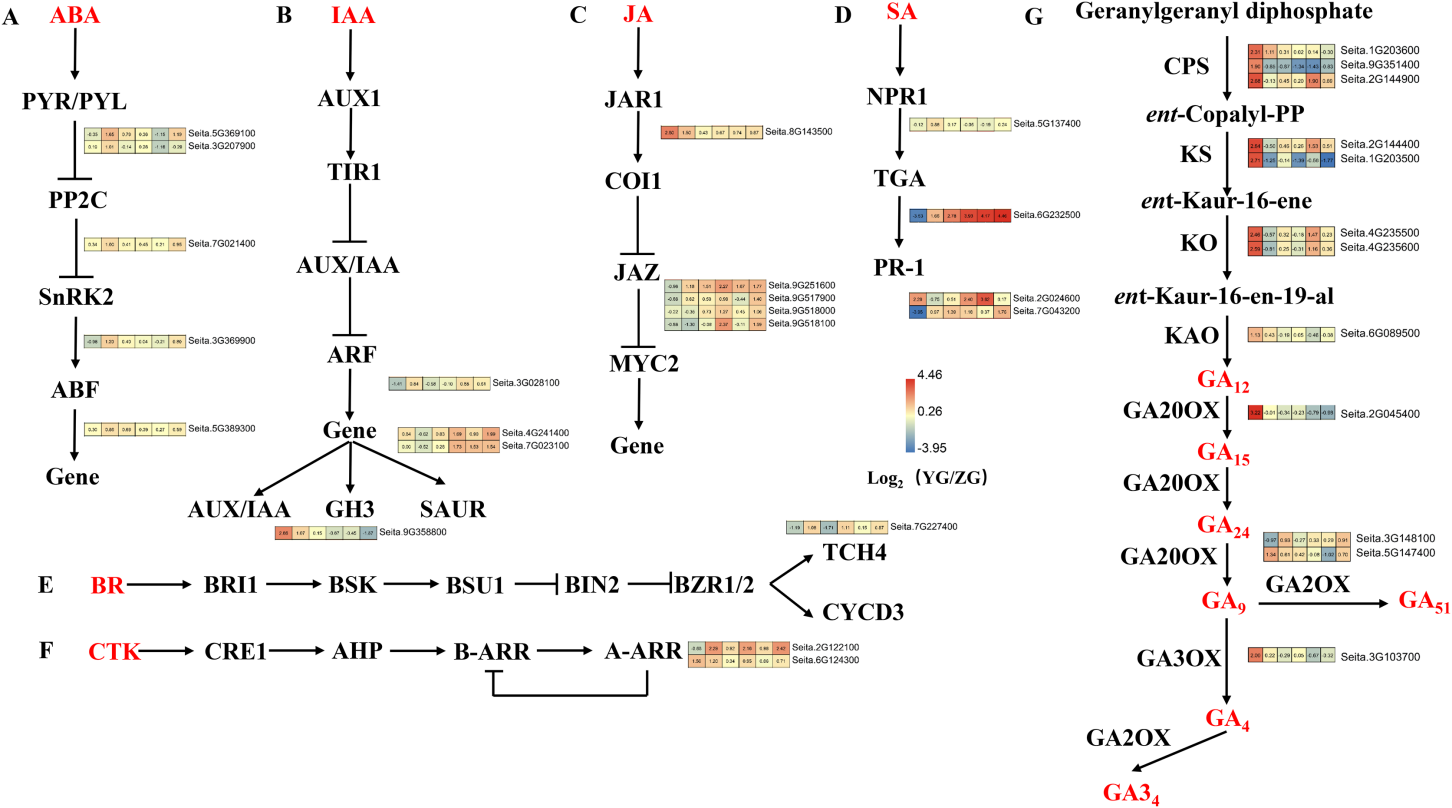
Heatmap analysis of genes associated with plant hormone signal transduction and GA biosynthesis during grain filling in both assessed accessions. The value of Log2 (FPKM in YG/FPKM in ZG) is represented using color depth; red and blue represent the upregulated and downregulated YG genes compared to those in ZG, respectively.

Upregulated DEGs in YG were enriched in the “plant hormone signal transduction” pathway at grain filling stages T2 and T4 (Fig. 6). Five DEGs related to abscisic acid (ABA) signal transduction were identified, including two *PYR/PYL*, one *PP2C*, one *SnRK*, and one *ABF* gene (Fig. 6A). These genes were highly expressed at most stages of the grain filling process of the YG accession, especially at T2 stage. Four DEGs related to the auxin (IAA) signal transduction pathway were identified, including one *ARF*, two *SAUR* and one *GH3*. Most of these genes were highly expressed in YG at different grain filling stages (Fig. 6B). Five jasmonic acid (JA) signal transduction-related DEGs (one *JAR1* and four *JAZ* genes) were also identified. Compared with the ZG accession, *JAR1* had a significantly higher expression during the early stages, however, the expression levels of 4 *JAZ* genes were lower in YG during the early stages but increased rapidly during the late stages (Fig. 6C). In addition, 4 DEGs related to salicylic acid (SA) signal transduction were upregulated at most grain filling stages in YG (Fig. 6D). Notably, *TGA* was strongly upregulated from grain filling stages T2 to T6 in YG compared to ZG. Moreover, brassinoid steroids (BR) and cytokinin (CK) signal transduction downstream genes had higher levels of expression at most stages in YG when compared to ZG (Figs. 6E, 6F).

### DEGs related to the GA biosynthesis pathway

The Diterpenoid synthesis pathway is a key pathway for GA biosynthesis. This pathway was significantly enriched in the upregulated DEGs in YG at both the preparation and initial stage of grain filling (T1 and T2). Seven gene families consisting of 12 genes involved in GA biosynthesis were identified as part of this analysis (Fig. 6G). GA biosynthesis genes in YG had a significantly higher expression than they did in ZG. In YG, the *CPS*, *KS*, *KAO*, *GA20OX*, *GA2OX*, and *GA3OX* encoded by loci Seita.9G351400, Seita.1G203500, Seita.6G089500, Seita.2G045400, Seita.5G147400, and Seita.3G103700, respectively, were all significantly upregulated at the T1 stage of grain filling. The *CPS* transcript encoded by loci Seita.1G203600 was significantly upregulated at grain filling stages T1 and T2. Similarly, two *KO*, one *CPS*, and one *KS* gene, encoded by Seita.4G235500, Seita.4G235600, Seita.2G144900, and Seita.2G144400, respectively, were significantly upregulated at the T1 and T4 stages of grain filling. The *GA2OX* encoded by Seita.3G148100 loci were significantly upregulated at grain filling stages T2 and T6.

### DEGs related to photosynthesis and the photosynthesis-antenna protein pathway

**Figure 7:**
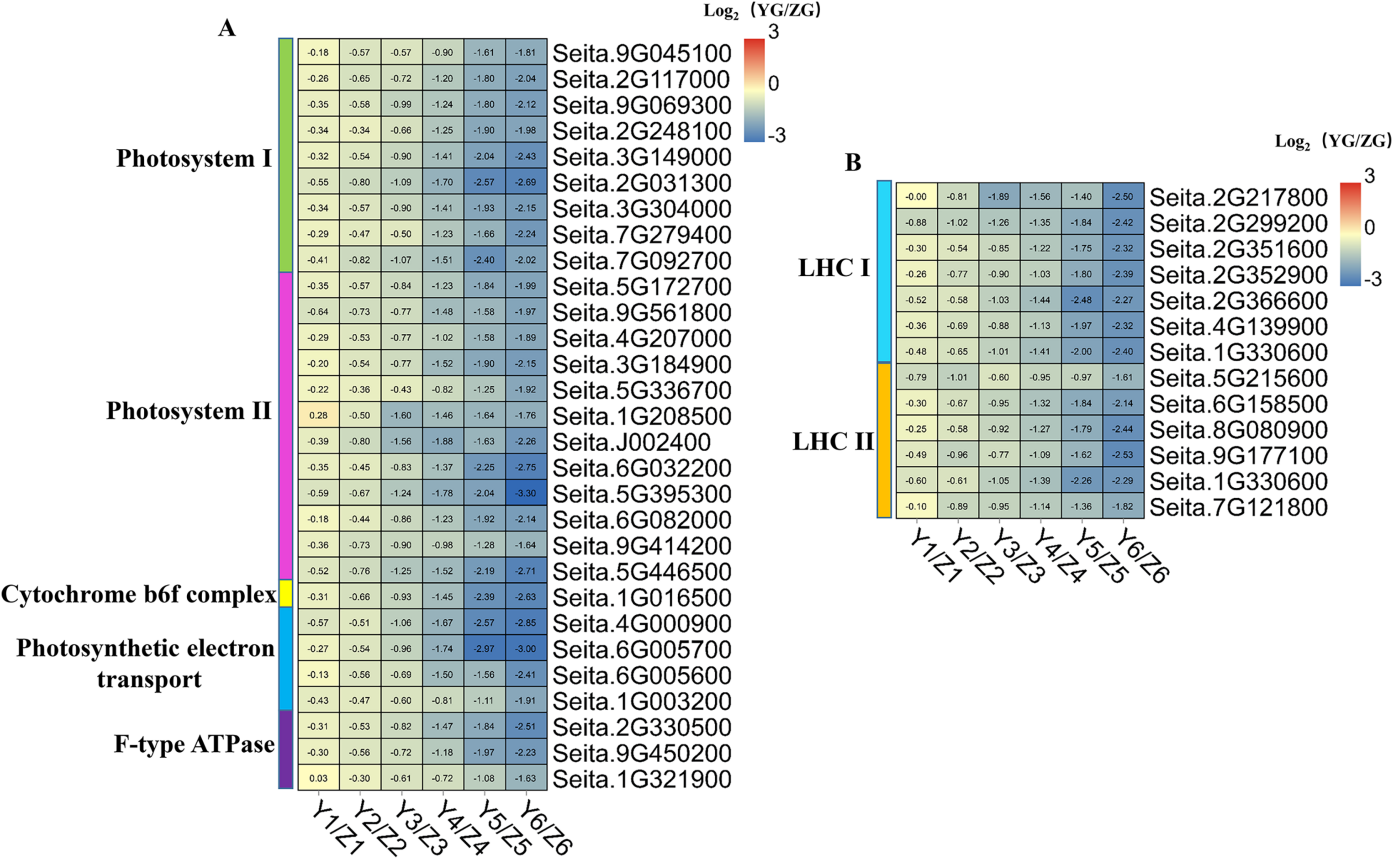
(A–B) Heatmap analysis of genes associated with photosynthesis and photosynthesis-antenna protein during grain filling in both assessed accessions. The value of Log2 (FPKM in YG/FPKM in ZG) is represented using color depth; red and blue represent the upregulated and downregulated YG genes compared to those in ZG, respectively.

The expression levels of genes involved in photosynthesis decreased during grain filling in both accessions (Fig. 2C). Besides, DEGs associated with photosynthesis and photosynthesis-antenna protein which in the YG accession had a significantly lower expression than they did in the ZG accession at the middle and late stages (Fig. 7).

We identified 29 DEGs associated with photosynthesis: nine involved in photosystem I, 12 in photosystem II, one in the cytochrome b6f complex, four in photosynthetic electron transport, and three associated with F-type ATPase function (Fig. 7). Thirteen DEGs associated with the photosynthesis-antenna protein were identified: seven related to LHC I and six to LHC II. Notably, all the DEGs associated with photosynthesis and the photosynthesis-antenna protein were highly expressed in the ZG accession, compared to the degree of their expression in the YG accession, especially during the middle and late stages. This phenomenon suggested that ZG could maintain a relatively high kernel photosynthetic capacity during grain filling compared to YG.

### qRT-PCR verification of RNA-seq gene expression data

**Figure 8:**
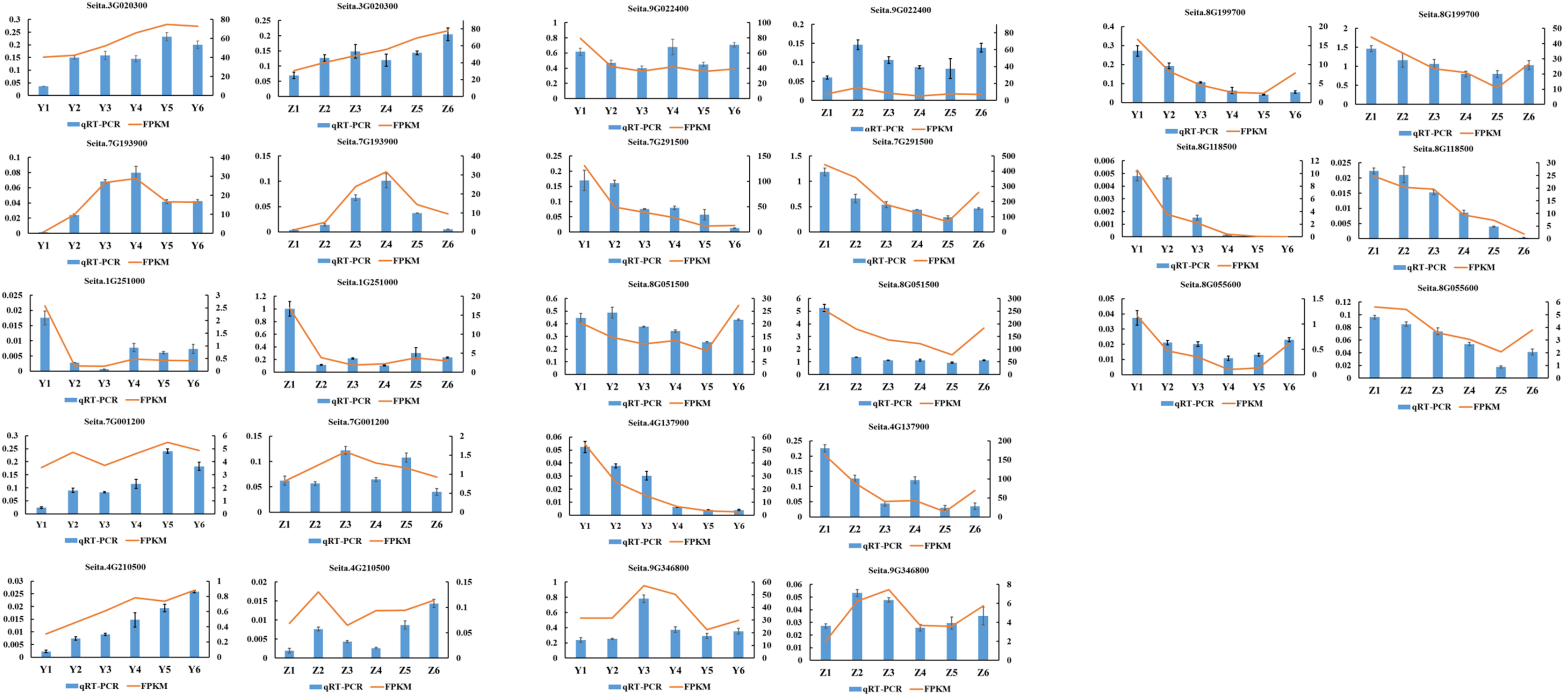
Quantitative real-time PCR (qRT-PCR) validation and RNA-seq data of 13 selected DEGs. Data shown were the mean of three independent repeated experiments ± standard deviation. Error bars represent standard deviations from three independent biological replicates.

A total of 13 randomly selected DEGs (Seita.3G020300, Seita.7G193900, Seita.1G251000, Seita.7G001200, Seita.4G210500, Seita.8G118500, Seita.8G055600, Seita.9G022400, Seita.7G291500, Seita.8G051500, Seita.4G137900, Seita.9G346800 and Seita.8G199700) were used to confirm the validity of the sequencing data. The results indicated that the expression pattern of all genes in the qRT-PCR assays were essentially consistent with the sequencing data, indicating that the RNA-seq analysis was reliable (Fig. 8).

## Discussion

Though modern cereal varieties, such as those of rice, have large panicles and numerous spikelets, their yield remains poor because of slow and incomplete grain filling (Sekhar et al., 2015; Das et al., 2018). In this study, ZG had a significantly higher grain weight per panicle than YG at early developmental stages because of its larger panicles. However, the weight of YG panicles increased rapidly and gradually overtook that of ZG panicles during the middle stages of the grain filling process, indicating that YG has a faster grain filling rate and more filled grains than ZG in the North China summer-sowing region. In this study, genes involved in photosynthesis, metabolic pathways, and phenylpropanoid biosynthesis were downregulated, while those related to peroxisome function, purine metabolism, and zeatin biosynthesis were upregulated during grain filling in both accessions. We identified most DEGs during the early stages in both accessions, a finding that suggests that dramatic gene expression changes occur early in the grain filling process.

ABC transporters have been proven to be widely involved in the transportation of secondary plant metabolites (Verrier et al., 2008). *TaABCC3* could promote grain formation in wheat. Its inhibition by virus-induced gene silencing (VIGS) reduced the grain number (Walter et al., 2015). Similarly, transgenic wheat lines whose *TaABCC13* expression is silenced have defective spike development (Bhati et al., 2016). The overexpression of *OsABCG18* significantly increased the levels of cytokinins in the shoot and improved grain yield in rice (Zhao et al., 2019). In our study, a number of ABC transporter-related DEGs were significantly upregulated in their expression level at the T5 and T6 stages of grain filling in YG compared to ZG. They included 3 ABC transporter B family members and 4 ABC transporter C family members. Compared with YG accession, the majority of ABC transporters showed higher level of expression during the early stages while presented a lower expression during the late stages of grain filling in the ZG accession, indicating that ABC transporters may play different roles at different grain filling stages in the two assessed accessions.

Phytohormones are small-molecule chemicals that potentially regulate kernel development and grain filling in crops. Six vital plant hormone signal transduction pathways associated with the DEGs were identified. The ABA signaling pathway is a primary grain filling regulator (Zhang et al., 2009). The key DEGs from the ABA signal transduction pathway were *PYR/PYL*, *ABF*, *SnRK2*, and *PP2C*. All were highly expressed in YG compared to ZG at most stages of the grain filling process, especially at the T2 stage. Auxins regulate panicle and spikelet development in rice. They can induce the rapid and transient expression of some genes, including *ARF* genes and primary auxin response genes such as *Aux/IAA, GH3*, and *SAUR* (Donato, Campbell & Franklin, 2012). The expression level of *SAUR* in YG was significantly higher than in ZG post the T3 stage of sampling. *GH3* was highly expressed in YG during the early of panicle development. Similarly, some key node genes in the CK, JA, SA, and BR signal transduction pathways were highly expressed in YG compared to ZG at most stages of the grain filling process. Previous studies have postulated that increasing the level of endogenous GA or spraying exogenous GA enhances the grain development process (Shi et al., 2020; Yang et al., 2011). The upregulated DEGs in YG were associated with GA biosynthesis (diterpenoids biosynthesis) at the preparation and initial stage of the grain filling process. Eleven DEGs involved in GA biosynthesis were identified. The expression level of these genes was significantly higher in YG than in ZG at both the preparation and initial stage of the grain filling process, suggesting that the rate of GA biosynthesis is positively correlated with grain filling in YG.

Though recent studies postulate that panicle/ear photosynthesis contributes to grain filling in C_3_ cereals (Tambussi et al., 2007; Maydup et al., 2012; Sanchez-Bragado et al., 2020), its role in C_4_ cereals remains unclear. In this study, STEM analysis revealed that photosynthesis-related genes were decreased across the grain development process in both accessions. We identified 29 and 13 DEGs associated with photosystem and photosynthesis-antenna proteins in both accessions, respectively. Their expression levels in YG were significantly lower than in ZG during the middle and late of grain filling. Thus, we hypothesize that the longer maturity period and slower chlorophyll degradation lead to the higher photosynthetic capacity of ZG grains.

In this study, kernels of the two assessed accessions of the same age were used for RNA-seq. However, since ZG has a longer kernel maturity period than YG, the differential expression of some genes may be due to the different developmental stages of the two assessed accessions when sampled for the transcriptomic analyses. Moreover, due to the different genetic backgrounds of the two assessed accessions, some DEGs identified in kernels may not be related to differences in grain filling. Further research is needed to verify these hypotheses.

This study reveals the transcriptome commonalities and differences between two foxtail millet accessions during grain filling at six distinct developmental stages. We show that genes of ABC transporters and genes involved in plant hormone signal transduction and the GA biosynthesis pathway are significantly upregulated in the YG, compared to the ZG accession, accounting for the rapid grain filling observed in the YG accession. As a whole, this study deciphers the putative molecular mechanisms of grain filling in foxtail millet as a reference for improving the yield potential of foxtail millet with large panicles and numerous spikelets in the North China summer-sowing region.

## Supplemental Information

10.7717/peerj.12968/supp-1 Supplemental Information 1 Primers used for qRT-PCR in this study.

10.7717/peerj.12968/supp-2 Supplemental Information 2 Overview of the RNA-seq data.

10.7717/peerj.12968/supp-3 Supplemental Information 3 GO terms enrichment of each comparison in YG.

10.7717/peerj.12968/supp-4 Supplemental Information 4 GO terms enrichment of each comparison in ZG.

10.7717/peerj.12968/supp-5 Supplemental Information 5 GO terms enrichment of up-regulated and down-regulated DEGs in YG compared with ZG.

10.7717/peerj.12968/supp-6 Supplemental Information 6 Raw data for qRT-PCR.

10.7717/peerj.12968/supp-7 Supplemental Information 7 Raw data for Figure 1.

10.7717/peerj.12968/supp-8 Supplemental Information 8 Unique DEGs were identified between YG and ZG.

## Additional Information and Declarations

### Data Availability

The following information was supplied regarding data availability: The raw sequencing data are available at the National Center for Biotechnology Information (NCBI): PRJNA721299; YG: SAMN18713804; ZG: SAMN18713832.
