# Supplementary material for: Comparative transcriptome analysis provides insights into grain filling commonalities and differences between foxtail millet [Setaria italica (L.) P. Beauv.] varieties with different panicle types
Source: PeerJ. 2022 Feb 18;10:e12968. doi: 10.7717/peerj.12968 (PMC8860066; doi:10.7717/peerj.12968)
Supplement: Supplemental Information 1 [file peerj-10-12968-s001.docx]

**Table S1: Primers used for qRT-PCR in this study.**

| **Primer Names** | **Primer Sequences (5′–3′)** |
| --- | --- |
| *SiACTIN7-F* | 5′- GGCAAACAGGGAGAAGATGA-3 |
| *SiACTIN7-R* | 5′- GAGGTTGTCGGTAAGGTCACG-3′ |
| *Seita.3G020300-F* | 5′-TCTGTTCCTCTGTGGGGACT-3′ |
| *Seita.3G020300-R* | 5′-CATGAATGGCTGTTGGGCAC-3′ |
| *Seita.7G193900-F* | 5′-ATCAACGAGGAGGTCAGGGA-3′ |
| *Seita.7G193900-R* | 5′-AAGCCAGTCTCTTCCACTGC-3′ |
| *Seita.1G251000-F* | 5′-GAACGAGAAGGCGAAAGCAG-3′ |
| *Seita.1G251000-R* | 5′-GCTCTCTTCTCTTCAGCCCG-3′ |
| *Seita.7G001200-F* | 5′-GAAGCCAGCAGTAGAGCGAT-3′ |
| *Seita.7G001200-R* | 5′-CAGCCGACCACCAAGGTTAT-3′ |
| *Seita.4G210500-F* | 5′-CTCCGCTCTTCGATGGAGTC-3′ |
| *Seita.4G210500-R* | 5′-GCTTCATTATGGCAAGGCCG-3′ |
| *Seita.9G022400-F* | 5′-GTGCAGTTGTTGTCCGCAAT-3′ |
| *Seita.9G022400-R* | 5′-AGCACCAAAACACATTGGGC-3′ |
| *Seita.7G291500-F* | 5′-GCGTGTGTGAGAGTGTGAGA-3′ |
| *Seita.7G291500-R* | 5′-GACGACGGATCACGTACGAA-3′ |
| *Seita.8G051500-F* | 5′-GGATTGTTGATGATGCCGTGG-3′ |
| *Seita.8G051500-R* | 5′-TCAGCTGCTTCCATTAGAGCC-3′ |
| *Seita.4G137900-F* | 5′-CCTGGGTATGGAGGAGGCTA-3′ |
| *Seita.4G137900-R* | 5′-GCAGACATCACTTCCACGCA-3′ |
| *Seita.9G346800-F* | 5′-GGAGGATAGGGCTGAGGGAA-3′ |
| *Seita.9G346800-R* | 5′-CCGAACTCGATCAACCTGCT-3′ |
| *Seita.8G199700-F* | 5′-GCCCACACAAACTCTTCACG-3′ |
| *Seita.8G199700-R* | 5′-CGACCAATCCTTCGCTCAGT-3′ |
| *Seita.8G118500-F* | 5′-AACGCCGAAGGCAAGAAGTA-3′ |
| *Seita.8G118500-R* | 5′-GGTTCATTCCCTCGACAGCA-3′ |
| *Seita.8G055600-F* | 5′-ATCTTTGGCCTTTGGGTGGT-3′ |
| *Seita.8G055600-R* | 5′-AGCGAACCATCATACAGCGG-3′ |
