# Supplementary material for: Comparative transcriptome analysis provides insights into grain filling commonalities and differences between foxtail millet [Setaria italica (L.) P. Beauv.] varieties with different panicle types
Source: PeerJ. 2022 Feb 18;10:e12968. doi: 10.7717/peerj.12968 (PMC8860066; doi:10.7717/peerj.12968)
Supplement: Supplemental Information 2 [file peerj-10-12968-s002.docx]

**Table S2. Overview of the RNA-seq data**

| **Sample** | **Raw Reads (M)** | **Clean Reads (M)** | **GC Content (%)** | **Q30 (%)** | **Mapped Reads (M (%))** | **Unique Mapped Reads (M (%))** | **Multiple Mapped Reads (M (%))** |
| --- | --- | --- | --- | --- | --- | --- | --- |
| Y11 | 60.54M | 59.35M | 52.49% | 94.89% | 94.43% | 92.08% | 2.35% |
| Y12 | 56.64M | 55.57M | 52.73% | 95.14% | 95.70% | 93.29% | 2.40% |
| Y13 | 52.32M | 51.22M | 53.01% | 95.40% | 93.91% | 91.56% | 2.35% |
| Y21 | 55.21M | 54.06M | 52.28% | 95.34% | 96.66% | 92.50% | 4.15% |
| Y22 | 52.67M | 51.54M | 52.96% | 95.35% | 95.96% | 91.84% | 4.13% |
| Y23 | 52.51M | 51.35M | 52.72% | 95.30% | 96.76% | 92.18% | 4.58% |
| Y31 | 52.22M | 51.22M | 53.25% | 95.44% | 95.43% | 90.59% | 4.84% |
| Y32 | 52.17M | 51.36M | 53.40% | 95.90% | 96.48% | 91.31% | 5.17% |
| Y33 | 46.04M | 45.27M | 53.30% | 95.70% | 96.73% | 91.85% | 4.88% |
| Y41 | 46.31M | 45.56M | 53.37% | 95.83% | 94.05% | 88.83% | 5.22% |
| Y42 | 48.39M | 47.52M | 53.86% | 95.75% | 96.14% | 91.38% | 4.77% |
| Y43 | 45.53M | 44.71M | 53.71% | 95.67% | 95.97% | 90.81% | 5.16% |
| Y51 | 51.27M | 50.37M | 53.89% | 95.76% | 93.92% | 87.38% | 6.53% |
| Y52 | 44.79M | 44.05M | 53.24% | 95.69% | 86.04% | 82.86% | 3.18% |
| Y53 | 48.49M | 47.45M | 52.88% | 95.07% | 93.01% | 87.83% | 5.18% |
| Y61 | 52.89M | 51.83M | 53.26% | 95.39% | 89.72% | 84.46% | 5.25% |
| Y62 | 44.79M | 43.82M | 53.43% | 95.05% | 89.75% | 86.79% | 2.95% |
| Y63 | 42.02M | 41.16M | 53.77% | 95.48% | 89.94% | 83.86% | 6.08% |
| Z11 | 57.88M | 56.72M | 52.66% | 94.81% | 96.94% | 94.30% | 2.64% |
| Z12 | 56.29M | 55.28M | 52.66% | 95.17% | 96.85% | 94.17% | 2.69% |
| Z13 | 53.07M | 51.95M | 52.30% | 94.82% | 95.91% | 93.30% | 2.60% |
| Z21 | 55.49M | 54.38M | 52.32% | 95.10% | 96.03% | 92.79% | 3.24% |
| Z22 | 56.59M | 55.54M | 52.62% | 95.17% | 95.42% | 92.47% | 2.95% |
| Z23 | 57.00M | 55.92M | 52.81% | 95.04% | 95.55% | 91.89% | 3.66% |
| Z31 | 55.40M | 54.36M | 52.90% | 95.16% | 94.77% | 90.34% | 4.43% |
| Z32 | 51.83M | 50.66M | 52.81% | 94.90% | 96.02% | 91.84% | 4.18% |
| Z33 | 60.21M | 58.98M | 52.56% | 94.60% | 95.47% | 91.19% | 4.28% |
| Z41 | 57.73M | 56.76M | 53.15% | 94.98% | 96.06% | 91.67% | 4.39% |
| Z42 | 59.10M | 57.94M | 53.12% | 94.57% | 95.51% | 91.22% | 4.29% |
| Z43 | 60.22M | 59.11M | 53.36% | 94.90% | 95.74% | 91.03% | 4.71% |
| Z51 | 56.63M | 55.32M | 53.95% | 94.62% | 93.56% | 88.93% | 4.63% |
| Z52 | 50.60M | 49.34M | 53.37% | 94.38% | 94.08% | 89.33% | 4.75% |
| Z53 | 60.17M | 58.94M | 53.21% | 94.67% | 95.81% | 91.16% | 4.66% |
| Z61 | 57.98M | 56.78M | 53.31% | 94.61% | 93.08% | 88.08% | 4.99% |
| Z62 | 58.27M | 56.97M | 52.48% | 94.82% | 92.91% | 89.45% | 3.46% |
| Z63 | 61.88M | 60.75M | 52.26% | 95.16% | 91.81% | 89.02% | 2.79% |

Notes: Y/Z1, Y/Z2, Y/Z3, Y/Z4, Y/ZT5 and Y/Z6 stand for six developmental stages (1, 7, 14, 21, 28, and 35 DAA) during grain filling of YG and ZG. _1,_2 and _3 represent the three biological replicates. M represents million. GC Content represents the percentage of guanine and cytosine in the clean reads. Q30 represents the percentage of nucleotides with a quality value ≥ 30.
