# Supplementary figures and images for: Comparative transcriptome analysis provides insights into grain filling commonalities and differences between foxtail millet [Setaria italica (L.) P. Beauv.] varieties with different panicle types
Source: PeerJ. 2022 Feb 18;10:e12968. doi: 10.7717/peerj.12968 (PMC8860066; doi:10.7717/peerj.12968)

# Y2 vs Y1

GO Term

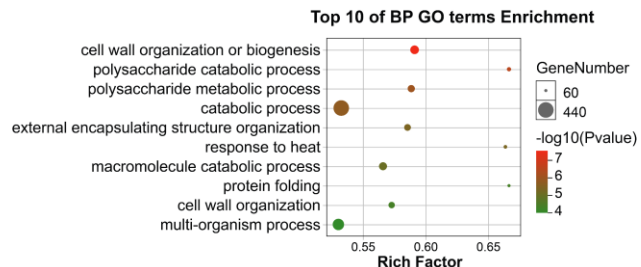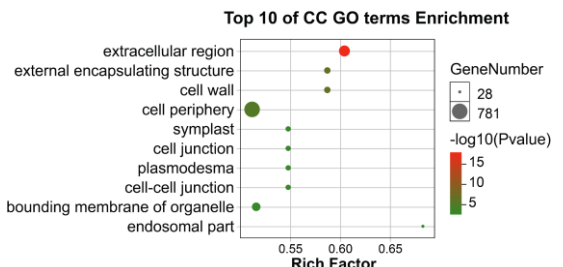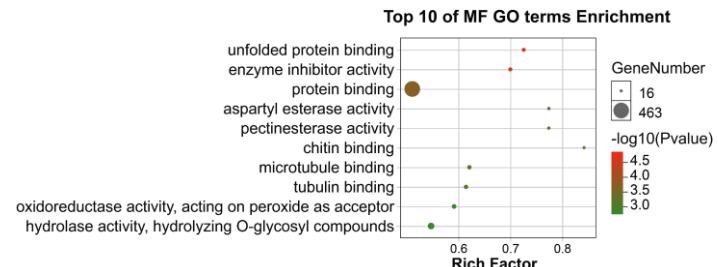

# Y3 vs Y2

GO Term

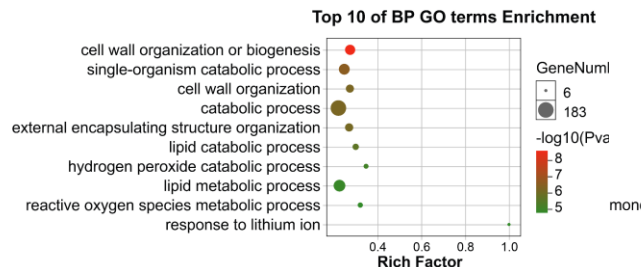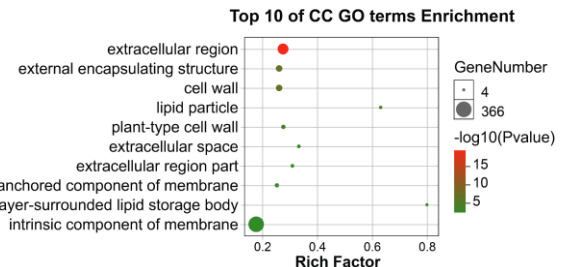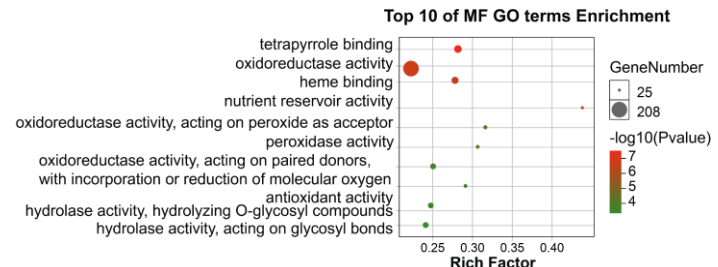

# Y4 vs Y3

GO Term

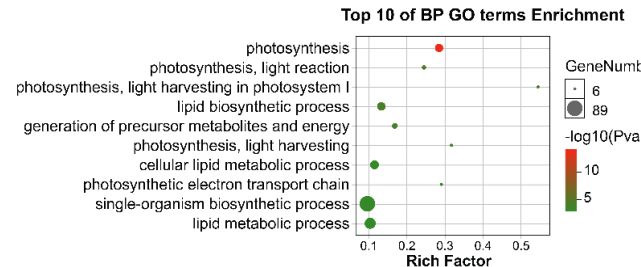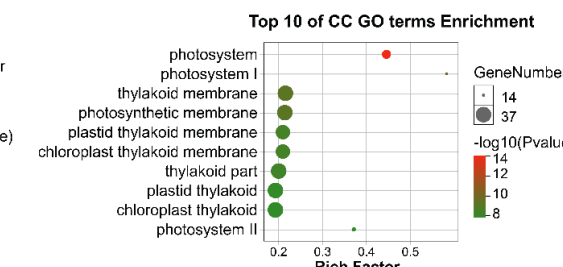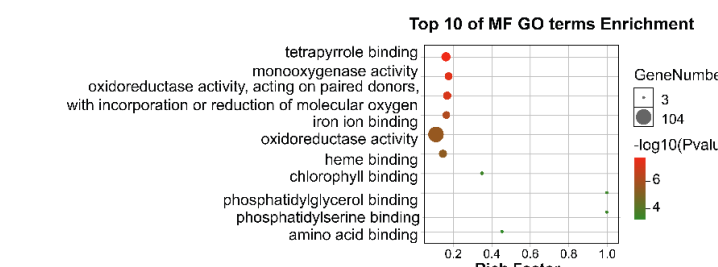

# Y5 vs Y4

GO Term

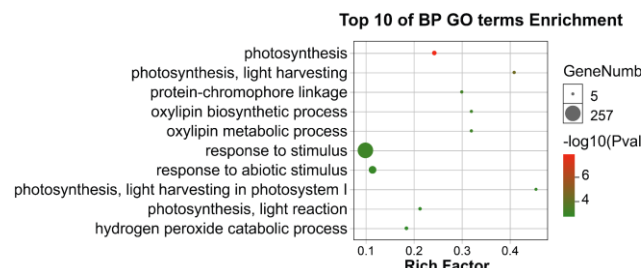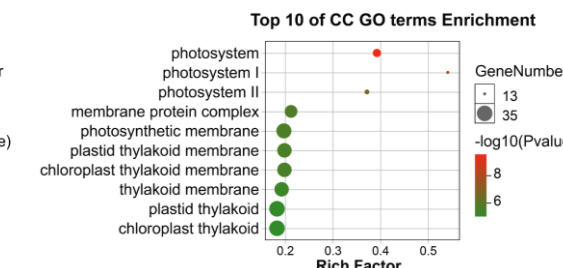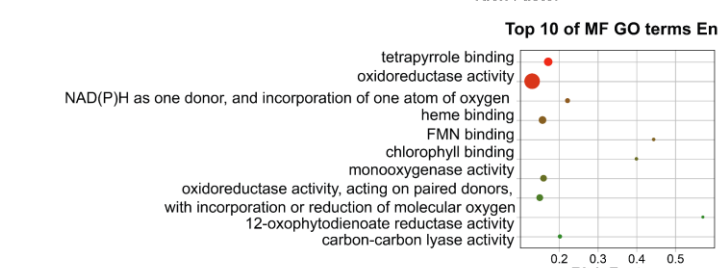

# Y6 vs Y5

GO Term

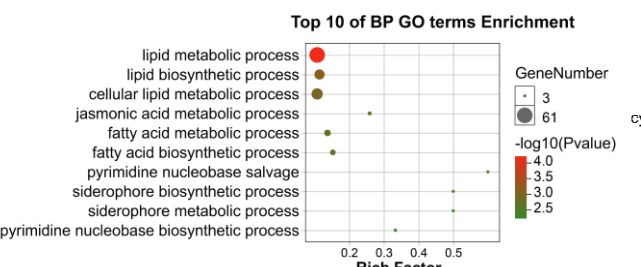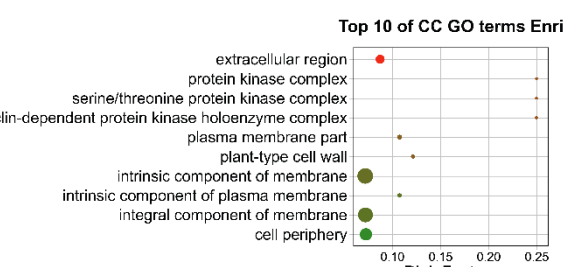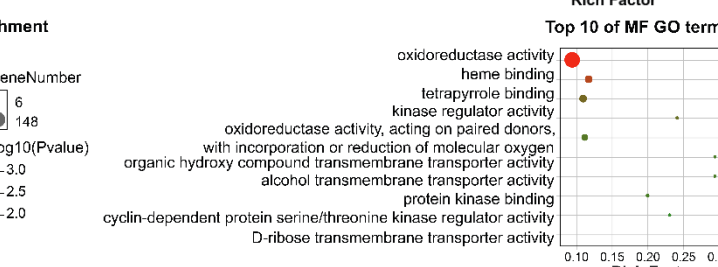

Supplement: Supplemental Information 3 [file peerj-10-12968-s003.pdf]

# Z2 vs Z1

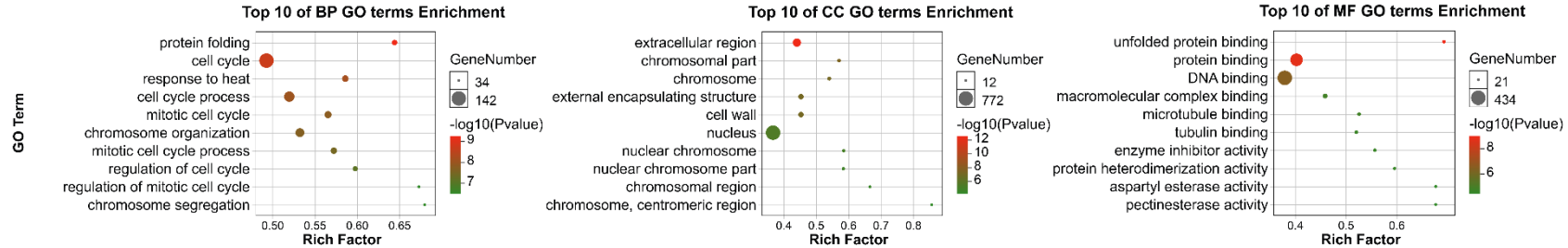

# Z3 vs Z2

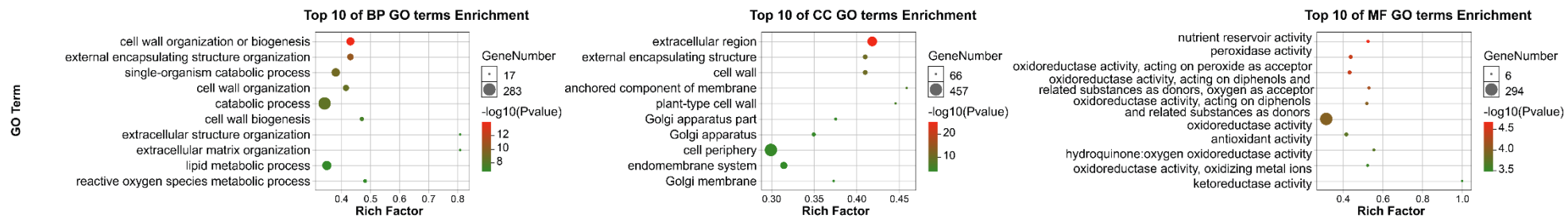

# Z4 vs Z3

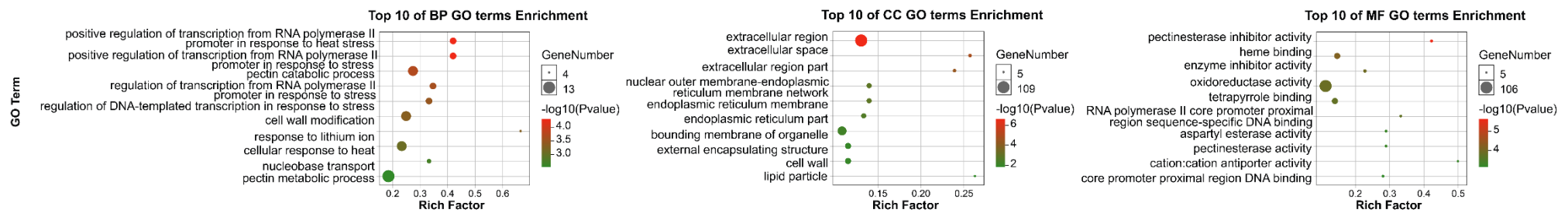

# Z5 vs Z4

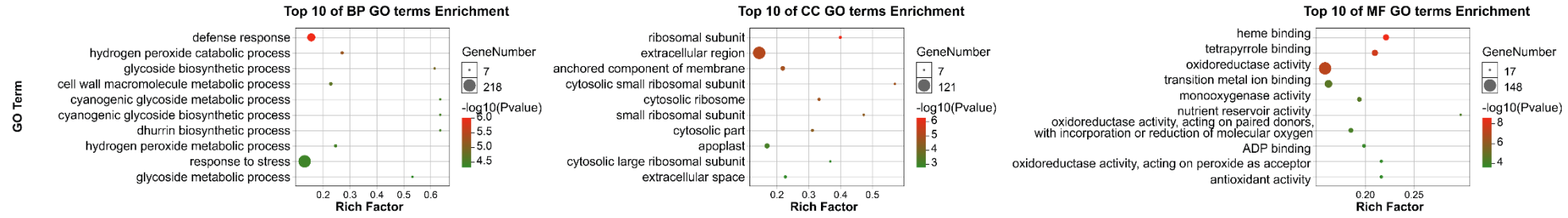

# Z6 vs Z5

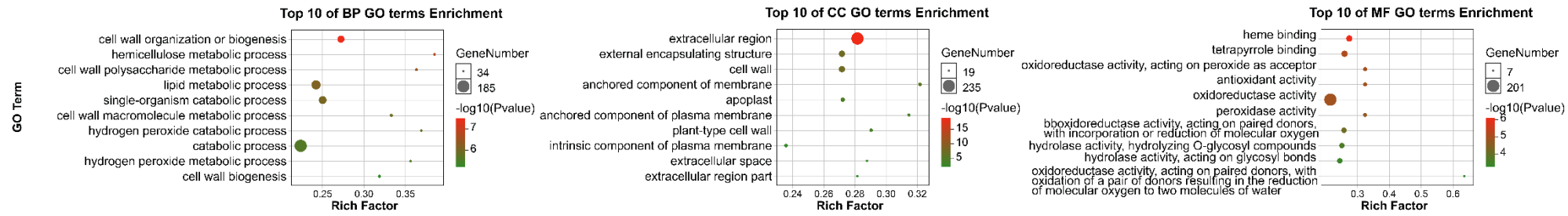

Supplement: Supplemental Information 4 [file peerj-10-12968-s004.pdf]

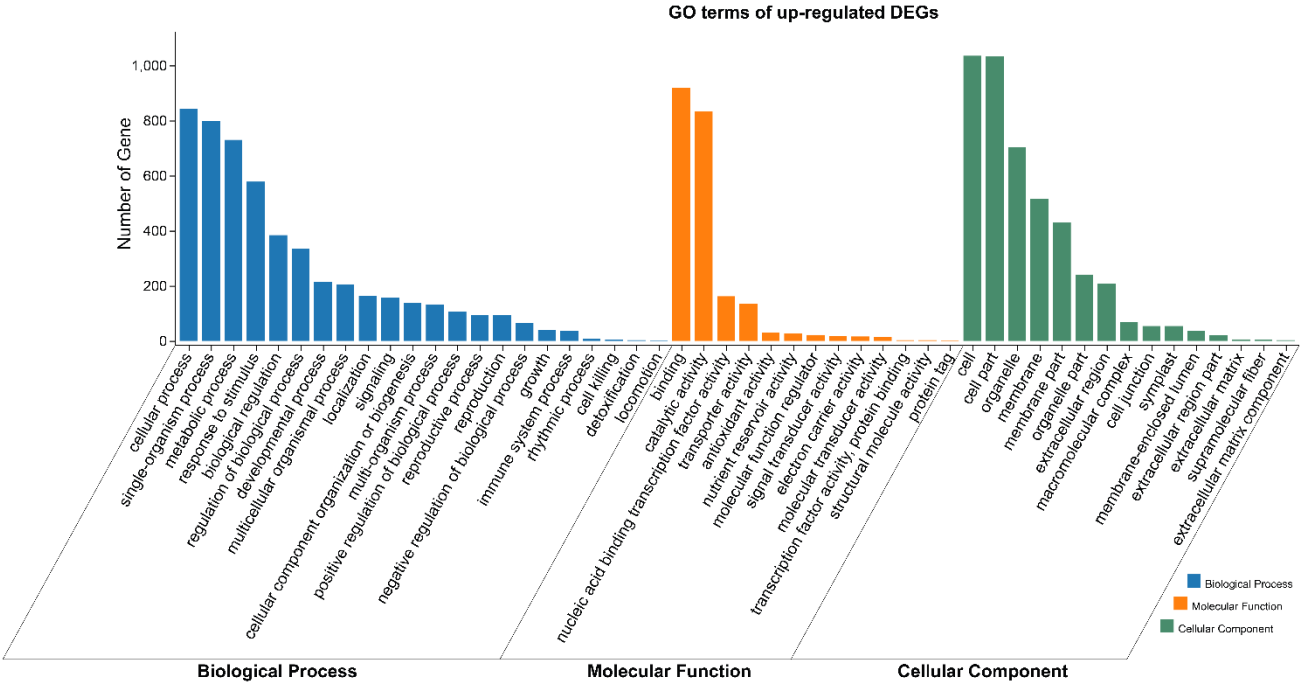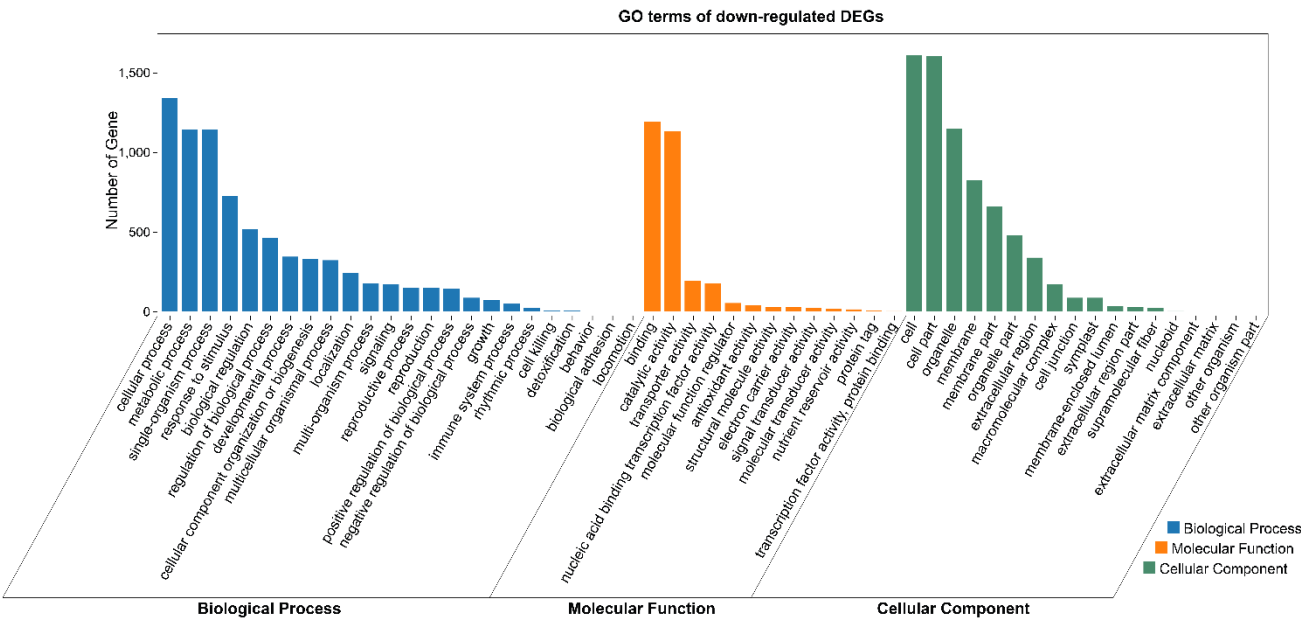

Supplement: Supplemental Information 5 [file peerj-10-12968-s005.pdf]
